# Ecotoxicological Impact of the Marine Toxin Palytoxin on the Micro-Crustacean *Artemia franciscana*

**DOI:** 10.3390/md20020081

**Published:** 2022-01-18

**Authors:** Federica Cavion, Marco Pelin, Cristina Ponti, Roberto Della Loggia, Aurelia Tubaro, Silvio Sosa

**Affiliations:** 1 Department of Life Sciences, University of Trieste, 34127 Trieste, Italy; federica.cavion@phd.units.it (F.C.); cponti@units.it (C.P.); tubaro@units.it (A.T.); ssosa@units.it (S.S.); 2 Department of Chemical and Pharmaceutical Sciences, University of Trieste, 34127 Trieste, Italy; rdellaloggia@units.it

**Keywords:** *Artemia*, palytoxin, ecotoxicology, mortality, oxidative stress, antioxidant enzyme activity

## Abstract

Palytoxin (PLTX) is a highly toxic polyether identified in various marine organisms, such as *Palythoa* soft corals, *Ostreopsis* dinoflagellates, and *Trichodesmium* cyanobacteria. In addition to adverse effects in humans, negative impacts on different marine organisms have been often described during *Ostreopsis* blooms and the concomitant presence of PLTX and its analogues. Considering the increasing frequency of *Ostreopsis* blooms due to global warming, PLTX was investigated for its effects on *Artemia franciscana,* a crustacean commonly used as a model organism for ecotoxicological studies. At concentrations comparable to those detected in culture media of *O.* cf. *ovata* (1.0–10.0 nM), PLTX significantly reduced cysts hatching and induced significant mortality of the organisms, both at larval and adult stages. Adults appeared to be the most sensitive developmental stage to PLTX: significant mortality was recorded after only 12 h of exposure to PLTX concentrations > 1.0 nM, with a 50% lethal concentration (LC_50_) of 2.3 nM (95% confidence interval = 1.2–4.7 nM). The toxic effects of PLTX toward *A. franciscana* adults seem to involve oxidative stress induction. Indeed, the toxin significantly increased ROS levels and altered the activity of the major antioxidant enzymes, in particular catalase and peroxidase, and marginally glutathione-S-transferase and superoxide dismutase. On the whole, these results indicate that environmentally relevant concentrations of PLTX could have a negative effect on *Artemia franciscana* population, suggesting its potential ecotoxicological impact at the marine level.

## 1. Introduction

**Figure 1:**
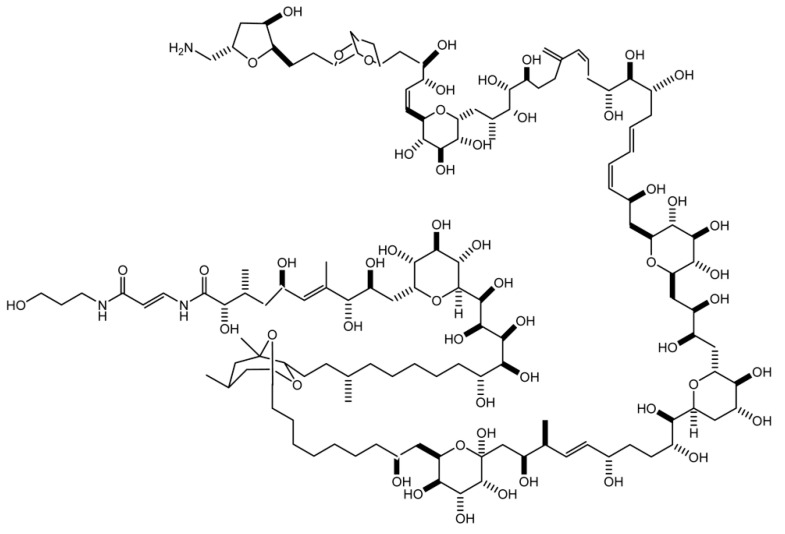
Structure of palytoxin.

The marine toxin palytoxin (PLTX; Figure 1) was firstly isolated in 1971, in Hawaii, from soft corals of the genus *Palythoa* [1]. The molecular target of PLTX is the sodium–potassium pump, expressed in all animal cells; the toxin binds to the pump, transforming it into a non-selective cationic channel. This modification allows the passive flow of ions driven by their electrochemical gradient, which causes an ionic disequilibrium in both excitable and non-excitable cells [2]. In the last few decades, this toxin and a series of its analogues have also been found in other marine organisms, such as soft corals of the genus *Zoanthus* [3], dinoflagellates of the genus *Ostreopsis* [4], and marine cyanobacteria of the genus *Trichodesmium* [5]. In temperate areas, such as the Mediterranean Sea and along the Southern European Atlantic coasts, PLTX and its analogues have been frequently detected during *Ostreopsis* blooms, in particular in *O.* cf. *ovata*. In the last few decades, these blooms appear to have increased in frequency, intensity, and expansion, probably due to (i) the eutrophication of coastal areas; (ii) the increased sea surface temperature, related to the global climate changes and favouring the geographical biomass distribution of *Ostreopsis* cells [6,7]; and (iii) the increased levels of floating materials in the sea, including seaweeds and marine plastic, allowing the attachment of epibenthic dinoflagellates and facilitating their diffusion [8,9,10].

Concomitantly to these events, a series of adverse effects in humans as well as negative impacts on different marine organisms have been documented, as summarized by several authors [11,12,13]. In contrast to safety issues for humans, the ecotoxicological potential of PLTX is still scarcely studied. Regarding this point, a very important aspect is the toxin impact on the marine ecosystem. In this context, cultures of PLTX-producing *O.* cf. *ovata* cells have mainly been used to investigate their effects on selected invertebrates. However, it should be underlined that some studies have been carried out without a complete qualitative and/or quantitative determination of the toxins in *Ostreopsis* cells or their culture media used in these studies [12,14,15,16,17]. In general, the results have showed the adverse effects of *O*. cf. *ovata* toward sea urchins, including reduced fertilization, impaired embryogenesis, and the correct development of first larval stages [14,15,16]. Other evidence in the bivalve *Mytilus galloprovincialis*, sampled during *O.* cf. *ovata* blooms, showed alterations of immunological and granulocyte lysosomal functions together with neurotoxic responses [13]. Furthermore, 7 and 14 days of exposure of *M. galloprovincialis* to cell cultures of *O.* cf. *ovata* in laboratory conditions revealed an impairment of the immune system, histological alterations in the digestive gland, and the increased activity of catalase, glutathione reductase, and glutathione peroxidase antioxidant enzymes [17]. In addition, Louzao et al. demonstrated a high in vitro cytotoxicity of purified PLTX toward *M. galloprovincialis* mantle and hepatopancreas cells: its half-maximal inhibitory concentration (IC_50_) on cells’ metabolic activity (alamarBlue assay) was equal to 18.23 nM and 16.17 nM, respectively [6]. An increased mortality after exposure to *O*. cf. *ovata* cells has also been observed in the copepod *Tigriopus fulvus* and the barnacle *Amphibalanus amphitrite* [12]. Moreover, a hydromethanol extract of *O.* cf. *ovata* (estimated PLTXs content = 358.8 ng/mL) induced lethal effects in the white shrimp *Litopenaeus vannamei* (moulting stage), associated with alterations of antioxidant enzymes activity, oxidative damage to lipids, the carbonylation of proteins, and the increased mRNA expression of immune genes [18].

Some *Artemia* species have been also used as model organisms to evaluate the ecotoxicological impact of *Ostreopsis* dinoflagellates and PLTX at the marine level. Using *A. salina* or non-identified *Artemia* species, the toxic effects induced by exposure to cultured *O. ovata* cells have been recorded by means of the increased mortality of nauplii [12,19] and adults [20]. Similarly, a study showed lethal effects toward nauplii for *O. siamensis* cells [21]. Other studies on *A. franciscana* showed that exposure to *O***.** cf. *ovata* cells induced a significant mortality in nauplii [22]. Lethal effects have also been recorded in *A. franciscana* nauplii exposed to *O.* cf. *ovata* cells lysates [15] and in *A. salina* nauplii exposed to a hydroethanol extract of *Palythoa caribaeorum* [23]. Other studies showed that *Artemia* nauplii are more sensitive to the lethal effect by the exposure to *O.* cf. *ovata* cells than the larvae of the copepods *Tigriopus fulvus* [12] and *Sarsamphiascus* cf. *propinquus* [22], or of the barnacle *Amphibalanus amphitrite* [12]. In general, these studies indicate the potential negative impact of *Ostreopsis* cells on *Artemia* organisms, ascribable to the presence of PLTXs. However, these findings were observed in *Artemia* species exposed to *Ostreopsis* sp. cells, with only a secondary extrapolation of their PLTX content, if available.

Considering the potential negative impact of *Ostreopsis* blooms and their toxins on the marine ecosystem, this study assessed the toxicity of PLTX toward *Artemia franciscana* as a marine zooplankton model organism. Apart from the lethal effect, the toxin was evaluated for its ability to induce oxidative stress through the quantitation of reactive oxygen species (ROS) and the activity of selected antioxidant enzymes (glutathione S-transferase, catalase, peroxidase, and superoxide dismutase), as well as by evaluating other parameters related to the organism motility.

## 2. Results

To assess the ecotoxicological impact of PLTX on *A. franciscana*, the toxin was initially evaluated for its effect on cysts hatching and mortality induction in the crustacean at two developmental stages (nauplii stage I and adults).

### 2.1. Effects of PLTX on A. franciscana Cysts Hatching

**Figure 2:**
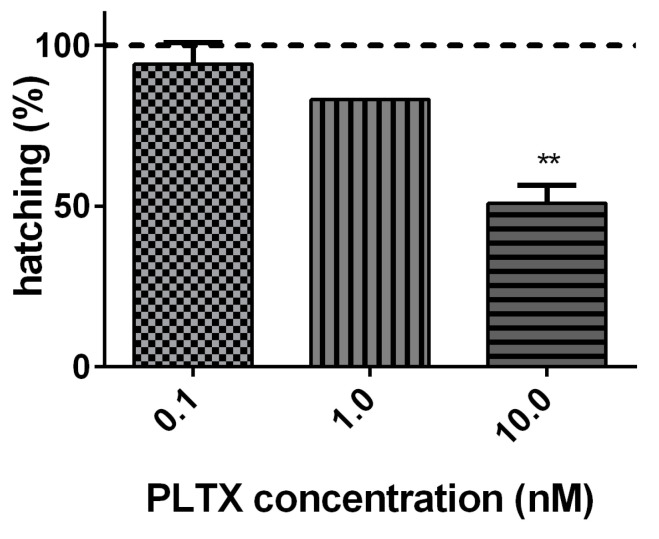
Percentage of *A. franciscana* cysts hatching after 96 h exposure to PLTX (0.1–10.0 nM) evaluated by stereomicroscope observation. Data are presented as % of free-hatched nauplii with respect to the total number of cysts exposed to the toxin and are expressed as mean ± SE of three experiments. Dashed line represents the hatching of cysts not exposed to the toxin (negative control). Statistical differences: ** *p* < 0.01 (one-way ANOVA and Bonferroni post-test).

The effect of PLTX (0.1, 1.0, and 10.0 nM) on *A. franciscana* cysts hatching was evaluated after 96 h of exposure. As compared to cysts not exposed to PLTX (negative control), the toxin induced a significant decrease of hatching (49%; *p* < 0.01) only at the highest concentration (10.0 nM) (Figure 2).

### 2.2. Effects of PLTX on A. franciscana Mortality

**Figure 3:**
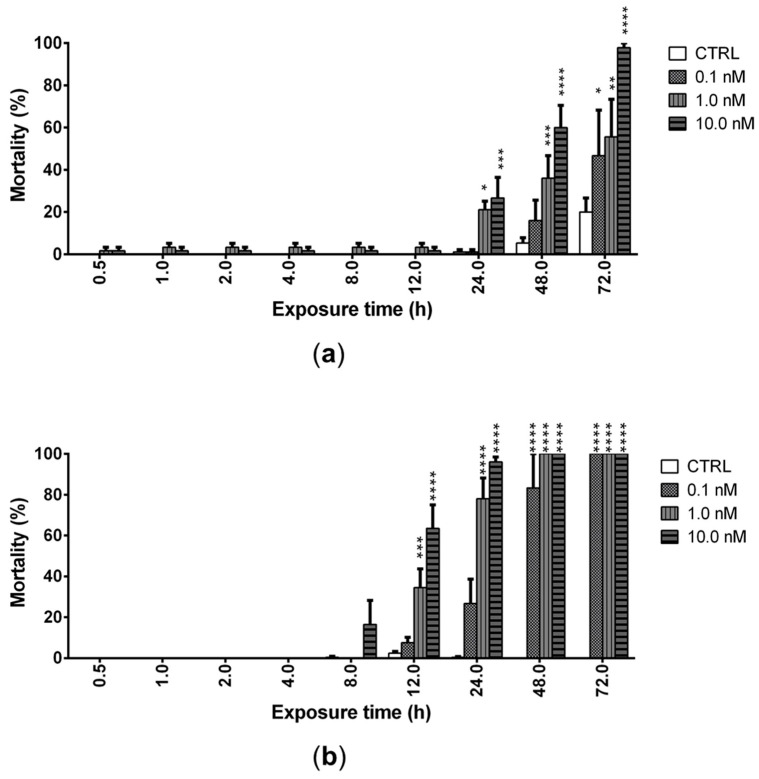
Mortality of *Artemia* nauplii (**a**) or adults (**b**) exposed to PLTX (0.1–10.0 nM) up to 72 h, evaluated by stereomicroscope. Data are presented as % of dead organisms with respect to the total number of animals and are expressed as mean ± SE of three experiments. Statistical differences: * *p* < 0.05; ** *p* < 0.01; *** *p* < 0.001; **** *p* < 0.0001 (two-way ANOVA and Bonferroni post-test).

The ability of PLTX (0.1, 1.0, and 10.0 nM) to induce mortality of *A. franciscana* nauplii stage I (24 h after hatching) and adults was evaluated after increasing times of exposure up to 72 h. PLTX induced a significant mortality of nauplii starting from 24 h of exposure to the concentrations of 1.0 and 10.0 nM. The lowest concentration (0.1 nM) induced a significant mortality only after 72 h of exposure (47%; *p* < 0.05), when the highest toxin concentration (10.0 nM) provoked 100% mortality of nauplii (Figure 3a). At this exposure time, the toxin concentration inducing 50% nauplii mortality (LC_50_) was 0.2 nM (95% confidence interval (C.I.) = 0.1–2.7 nM).

In adults, PLTX induced a significant mortality after only 12 h of exposure to the concentrations of 1.0 (35%; *p* < 0.001) and 10.0 nM (64%; *p* < 0.0001), with an LC_50_ of 2.3 nM (C.I. = 1.2–4.7 nM). The lowest toxin concentration (0.1 nM) induced a significant mortality after only 48 h of exposure (83%; *p* < 0.0001), whereas 100% mortality was induced by 72 h of exposure to all PLTX concentrations (Figure 3b).

These results suggest that *A. franciscana* adults are more sensitive than nauplii stage I to the toxic effects of PLTX, which is able to significantly increase adults’ mortality after only 12 h of exposure. Thus, the subsequent analyses were carried out on adults exposed to PLTX for 12 h.

### 2.3. Effect of PLTX on ROS Production in A. franciscana Adults

**Figure 4:**
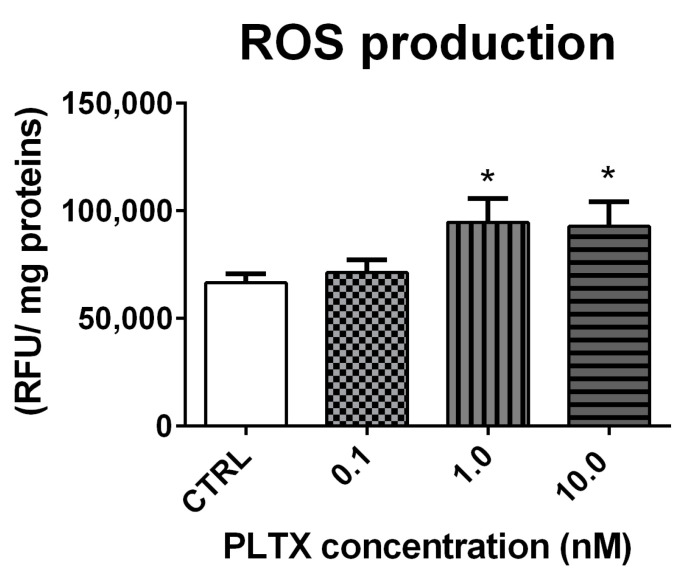
ROS production in *A. franciscana* adults after 12 h exposure to PLTX evaluated by the DCFDA fluorescence assay. Data are presented as relative fluorescent units normalized on milligrams of proteins in each sample and are expressed as mean ± SE of four experiments. Statistical differences: * *p* < 0.05 (Student’s *t*-test).

ROS production in *A. franciscana* adults exposed to PLTX for 12 h was evaluated by the DCFDA fluorescence assay. As compared to the negative control, the toxin induced a significant increase of ROS at the concentrations of 1.0 and 10.0 nM (42% and 39%, respectively; *p* < 0.05) (Figure 4).

### 2.4. Effect of PLTX on Antioxidant Enzymes Activity in A. franciscana Adults

**Figure 5:**
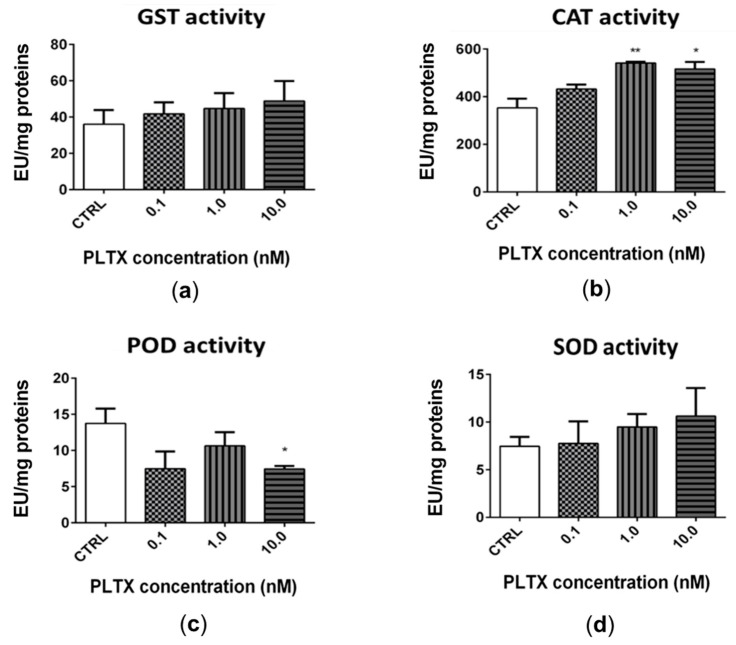
Activity of glutathione S-transferase (GST) (**a**), catalase (CAT) (**b**), peroxidase (POD) (**c**), and superoxide dismutase (SOD) (**d**) in *A. franciscana* adults after 12 h exposure to PLTX. Data are presented as enzymatic units (*EU*) normalized on milligrams of proteins in each sample and are expressed as mean ± SE of at least three experiments. Statistical differences: * *p* < 0.05; ** *p* < 0.01 (Student’s *t*-test).

The exposure of *A. franciscana* adults to PLTX for 12 h slightly altered the activity of glutathione S-transferase (GST), catalase (CAT), peroxidase (POD), and superoxide dismutase (SOD) (Figure 5). The most significant effect was recorded for CAT (Figure 5b), whose activity was significantly increased by exposure to 1.0 and 10.0 nM PLTX (53% and 46%, respectively; *p* < 0.01) (Figure 5b). The exposure of *A. franciscana* to PLTX also induced a slight but not significant increase of GST and SOD activities (Figure 5a,d). On the contrary, POD activity was significantly decreased (46%) only at the highest toxin concentration (10.0 nM; *p* < 0.05) (Figure 5c).

### 2.5. Effect of PLTX on Cholinesterase Activity in A. franciscana Adults

**Figure 6:**
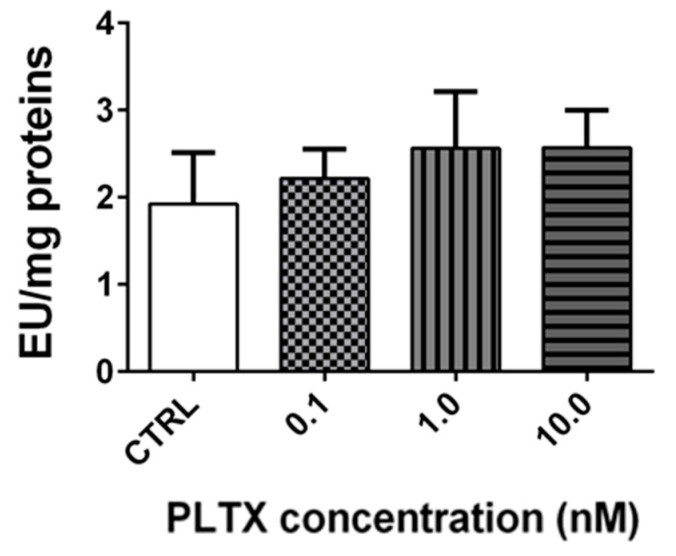
Cholinesterase activity in *A. franciscana* adults exposed to PLTX for 12 h. Data are presented as enzymatic units (*EU*) normalized on milligrams of proteins and expressed as mean ± SE of at least three experiments.

Stereomicroscopy observations revealed that the exposure of *A. franciscana* adults to PLTX reduced crustacean motility in a concentration-dependent manner (Video S1 in the Supplementary Materials). At the highest concentration (10.0 nM), *A. franciscana* motility was almost completely hampered. To verify whether this effect could be related to an altered cholinergic transmission, the activity of cholinesterase (ChE), an enzyme involved in the regulation of *Artemia* movement [24], was evaluated in adults exposed to the toxin for 12 h. However, the obtained results show that the enzyme activity was not significantly altered by PLTX (Figure 6).

## 3. Discussion

*Artemia* species are considered representative organisms of marine zooplankton and, therefore, they are widely used as organism models for ecotoxicological studies [25,26,27,28]. Indeed, *Artemia* species are present in natural and/or man-made hypersaline lakes, temporary desert ponds, coastal lagoons, saltern ponds, pools, and saltmarshes [29], with a wide geographic distribution, being found in 500 sites [29] spread over all the continents, except Antarctica [30]. The life cycle of species belonging to the genus *Artemia* includes 15 larval stages (nauplii), the juvenile stage, and the adult one. This genus includes many species with parthenogenic reproduction and six bisexual species [26]. The bisexual species have two reproductive strategies: ovoviparous reproduction, characterized by fertilized eggs developing free-swimming nauplii, or oviparous reproduction, characterized by encysted embryos production, hatching under favourable environmental conditions [29]. *Artemia* species are characterized by a high adaptability to environmental conditions, such as wide ranges of salinity (5–250 g/L) and temperature (6–35 °C) [26]. Furthermore, these crustaceans are characterized by a short life cycle, high fecundity, small body size, wide geographic distribution, and adaptability to a variety of nutrient resources, being non-selective filter feeders. In addition, *Artemia* spp. are an essential food source for numerous bird species in their natural habitat [31]. For these reasons, *Artemia* spp. are commonly used for aquatic ecotoxicological studies, in particular for the marine environment [25] and for bioaccumulation studies of substances along the food web [32].

On the basis of these considerations, *Artemia franciscana* was used to assess the potential ecotoxicological impact of PLTX at the marine level. Indeed, in addition to a series of adverse effects in humans ascribed to PLTX and/or its analogues during *Ostreopsis* blooms, negative impacts have been observed in different invertebrate and vertebrate marine organisms [33,34].

The toxin effects on *A. franciscana* were evaluated at concentrations (0.1–10.0 nM) comparable to those detected in culture media of *O.* cf. *ovata* cells [35]. The effects were initially evaluated on three developmental stages of *A. franciscana*: cysts (toxin effect on cysts hatching), nauplii stage I, and adults (organisms’ mortality induced by the toxin). The hatching assay, which is rapid, simple, and sensitive [36], showed that the exposure of *A. franciscana* cysts to PLTX for 96 h induced a significant hatching reduction only at the highest concentration (10.0 nM). Furthermore, the exposure of *A. franciscana* nauplii stage I and adults to the toxin up to 72 h induced a concentration- and time-dependent increase of mortality. In nauplii stage I, PLTX significantly increased the organisms’ mortality (<50%) starting from 24 h of exposure to the concentrations of 1.0 and 10.0 nM. This effect appears slightly lower than that recorded by Pezzolesi et al. [19] in *Artemia* sp. nauplii (species and nauplii stage not specified) exposed to PLTX for 24 h: a median lethal concentration (LC_50_) of 4.6 ng/mL (corresponding to around 1.7 nM PLTX) was determined. This different potency might be related to differences between the *Artemia* species and nauplii stage used. In particular, the variable inter-species sensitivity is considered as one of the possible factors influencing the response of *Artemia* crustaceans to numerous substances [26,37]. Pavaux et al. [22] recorded about 80% mortality of *A. franciscana* metanauplii (stages II–III) after 24 h of exposure to *O.* cf. *ovata* cells (4 cells/mL; PLTX equivalents content: 44 ± 17 pg/cell by UHPLC-UV-HRMS). However, it is difficult to compare the results of our study with those obtained by Pavaux and colleagues since the type and the amount of the individual toxins in *Ostreopsis* culture were not specified. Furthermore, it is known that the major toxin produced by cultures of *O.* cf. *ovata* is ovatoxin-a, PLTX being produced only in traces [19].

PLTX significantly increased mortality of *A. franciscana* adults after only 12 h of exposure to the concentrations of 1.0 and 10.0 nM, with an LC_50_ of 2.3 nM (C.I. = 1.2–4.7 nM). To the best of our knowledge, this is the first study assessing the toxic effects of purified PLTX on *Artemia* adults in comparison to nauplii, suggesting that adults are more sensitive to the toxin than larvae. This different sensitivity could be due to the incomplete development and functioning of the digestive tract in *Artemia* nauplii stage I [29], which could limit the toxin absorption. Indeed, Instar I larvae’s lack of critical anatomical formations (i.e., mouth and anus) limits the ingestion and the possible bioaccumulation of compounds which could not be absorbed through the external surface of *Artemia* due to their not-sufficient lipophilicity or small dimensions, such as PLTX. Our finding is in agreement with previous studies showing that *Artemia* stage I nauplii are more resistant than adults to some xenobiotics, such as inorganic chemical reagents and elements (copper) [38,39] or nanomaterials [40,41]. Notwithstanding, PLTX effects recorded by our study on *A. franciscana* cysts hatching and its lethality for adults, as well as the effects on nauplii observed by previous studies, suggest that seawater concentrations of PLTX during *Ostreopsis* blooms could negatively impact *Artemia* population in marine zooplankton.

As compared to nauplii stage I, the higher sensitivity of *A. franciscana* adults to PLTX prompted us to further investigate the toxin effects on adults after 12 h of exposure, the shortest exposure time at which PLTX induced a significant, but not complete, mortality. The main focus was directed toward oxidative stress, a phenomenon involved in the mechanism of PLTX toxicity exerted in both marine organisms’ cells [17,18] and human cells [42,43,44]. The exposure of *A. franciscana* adults to PLTX for 12 h significantly increased ROS production at the concentrations of 1.0 and 10.0 nM, inducing a significant mortality after the same exposure time. Therefore, it is conceivable that oxidative stress may be involved in the induction of mortality, knowing that ROS overproduction can damage proteins, lipids, and DNA [45] and is implicated in many cell processes, including apoptosis [46].

Oxidative stress induction by PLTX in *A. franciscana* is also confirmed by the slight alteration of the main antioxidant enzymes activity, specifically glutathione S-transferase (GST), catalase (CAT), peroxidase (POD), and superoxide dismutase (SOD). GST belongs to the phase II detoxification enzymes, and conjugates reduced glutathione (GSH) to a wide variety of electrophilic compounds [47]; it has also been identified at significant levels in *Artemia*, where it can bind a wide range of substrates [48], including xenobiotics and ROS. The first line of defence toward increased ROS levels in aquatic invertebrates is represented by SOD and CAT antioxidant enzymes [49,50]. Among peroxidases, glutathione peroxidase (GPx) has also been identified in *Artemia*, in particular at the larval stage [51]. In general, the major effects of PLTX on *A. franciscana* antioxidant enzymes involved CAT and POD. The exposure of *A. franciscana* to PLTX for 12 h increased CAT activity at the selected concentrations (1.0 and 10.0 nM) that also significantly increased ROS levels. On the other hand, the highest PLTX concentration (10.0 nM) significantly decreased POD activity. This effect can be explained by the complementary roles of CAT and POD (in particular GPx) in the detoxification of hydrogen peroxide (H_2_O_2_), with different affinities for the substrate and subcellular localizations: CAT is localized in the cytosol, while POD is localized in peroxisomes. Consequently, high levels of H_2_O_2_ are associated with an increased affinity for CAT, while low H_2_O_2_ levels are associated with a higher affinity for POD [52]. Thus, CAT and POD (in particular GPx) activity patterns are expected to vary across redox cycling compounds, depending on the nature, amount, and subcellular localization of the produced ROS [53]. An increased CAT activity associated with decreased GPx activity has already been recorded in other marine organisms exposed to the ROS inducer menadione, including the crustacean *Daphnia magna* [53], the mussel *Mytilus edulis* [54], and the fish *Oncorhynchus mykiss* [55].

Regarding GST and SOD activity in *A. franciscana* adults exposed to PLTX, despite a trend showing a slight concentration-dependent increased activity, this effect was not significant. An increased GST activity has been previously recorded in other aquatic organisms exposed to toxins different from PLTX, such as shrimps (*Litopenaeus vannamei*) exposed to cyanobacteria extracts containing microcystins, [56] and *Artemia salina* adults exposed to microcystin-LR or nodularin, in which conjugation with GSH has been shown as an initial detoxification mechanism [57]. On the contrary, the slight increase of GST activity in *A. franciscana* exposed to PLTX suggests a low induction of the GST detoxification system. A similar conclusion is suggested for SOD, whose activity was not influenced by the toxin, even at its highest concentration. Nonetheless, the recorded effects indicate that PLTX can perturbate the main antioxidant systems in *A. franciscana*, corroborating its ability to induce ROS production and oxidative stress. However, the low effect on these enzymes’ activity, especially on SOD and GST, suggests that these detoxification systems are not sufficiently activated to protect the crustacean from PLTX toxicity. In addition, the slight increase of these ROS-detoxification systems and the low, albeit significant, increase of ROS levels induced by the toxin suggest that oxidative stress might not be the primary and/or the only mechanism leading to *Artemia* death. Thus, we cannot exclude the possibility that other physiological perturbations may co-occur to induce the organism death.

Stereomicroscopy observations of *A. franciscana* exposed to PLTX showed a concentration-dependent decrease of adults’ motility, as previously noted in *A. salina* adults exposed to *O.* cf. *ovata* cells [20]. Hypothesizing a PLTX effect on the cholinergic transmission system, the activity of cholinesterase has been evaluated in adults exposed to PLTX, considering the role of the enzyme in maintaining the normal neuromuscular function. In fact, an increased level of the neurotransmitter acetylcholine in the synapses, consequent to cholinesterase enzymes inhibition by xenobiotics, can impair the neuromuscular functions [58]. For this reason, cholinesterase activity is considered a biomarker of toxicity in *Artemia* spp. as well as in other marine invertebrates [24,37]. However, cholinesterase activity was not influenced in *A. franciscana* adults exposed to PLTX, suggesting that the decreased crustacean motility is not related to an impaired enzyme activity by the toxin. However, it should be noted that the regulation of *Artemia* species’ locomotion is a complex biochemically-controlled phenomenon that cannot be attributed only to cholinesterase activity, even though it is one of the best characterized enzymes in these organisms. Therefore, we cannot exclude the possibility that other biochemical pathways could be involved in PLTX effects on the motility of *A. franciscana*. For instance, the reduced *A. franciscana* movements could be ascribed to an altered ion homeostasis involving the physiological control of fluid dynamics, as already hypothesized for *A. salina* adults exposed to PLTX analogues released by *O.* cf. *ovata* cells [20] and *A. salina* nauplii exposed to other toxins produced by the dinoflagellate *Prorocentrum lima* [59].

## 4. Conclusions

In the last few decades, *Ostreopsis* blooms recorded in temperate coastal waters have been frequently associated with adverse effects in humans and in different marine organisms, probably due to the production and release of PLTX and its analogues. Given the possible expansion of *Ostreopsis* spp. in terms of increasing blooms intensity and frequency as well as colonization of new areas due to the ongoing global warming, the impact of these dinoflagellates and their toxins needs to be assessed not only for humans but also for marine organisms. The present ecotoxicological study on *Artemia franciscana* showed that environmentally relevant PLTX concentrations induce detrimental effects on this micro-crustacean, being able to significantly reduce cysts hatching and to provoke the mortality of the organisms both at larval and adult stages. Adults turned out to be the most sensitive developmental stage of *A. franciscana* to PLTX, which induced oxidative stress. Indeed, the toxin significantly increased ROS levels and altered the activity of some of the major antioxidant enzymes, in particular catalase and peroxidase. Since *Artemia* species are members of the zooplankton community at the basis of the marine food web, a decrease of their population could also have a negative impact on organisms at a higher trophic level, and consequently on the marine biodiversity. However, further studies on other aquatic organisms are required for a complete understanding of the ecological impact of PLTX at the marine level.

## 5. Materials and Methods

### 5.1. Artemia franciscana

*Artemia franciscana* cysts were purchased from a commercial seller of aquatic products (Hobby; Garnelenhaus; Barsbüttel, Germany). The taxonomic identification of the species was previously confirmed by PCR sequencing [41]. *A. franciscana* was bred in seawater and prepared with 36 g/L of specific salts for marine organisms (Optimum Sea basic salt; Wave) at 25 °C under a 16:8 h light/dark cycle, except for hatching, during which a constant artificial light source was provided. Nauplii collected after 24 h from hatching (stage I) and the adults (collected after 21 days of culture) were used. Nauplii were fed three times a week, starting after 3 days from hatching, with liquid food (Hobby-Liquizell; Garnelenhaus; Barsbüttel, Germany) up to 10 days, and subsequently with solid food (Hobby-Mikrozell; Garnelenhaus; Barsbüttel, Germany).

### 5.2. Palytoxin and Experimental Design

Palytoxin (PLTX), isolated from *Palythoa tuberculosa* (purity > 95%), was purchased from Wako Pure Chemicals Industries, Ltd. (Osaka, Japan). The toxin, dissolved in ethanol and distilled water at a 1:1 ratio (*v*/*v*), was tested at the concentrations of 0.1, 1.0, and 10.0 nM with a final concentration of ethanol equal to 0.5%. This concentration of ethanol, which did not induce any sign of toxicity, was also included in the negative control (*A. franciscana* not exposed to the toxin; CTRL).

The experimental design consisted of a first step aimed at evaluating the effects of PLTX on *A. franciscana* at three developmental stages (cysts hatching and the mortality of larvae and adults), to determine the most sensitive one. The mortality test was based on a concentration- and time-dependent approach to determine the optimal exposure condition for elucidating the mechanism of PLTX toxicity on the most sensitive developmental stage, in the second step of the study.

### 5.3. Hatching Assay

Hatching assay was performed according to Migliore et al. [60]. Briefly, in each well of a 96-well plate, 10 cysts were suspended in 200 µL of seawater and exposed to PLTX (0.1, 1.0, and 10.0 nM). The number of nauplii was determined every 24 h, up to 96 h, under a stereomicroscope at a 3× magnification (Kyowa; Tokyo, Japan). At 96 h, hatching was calculated as the ratio between the number of free-hatched nauplii and the number of cysts and expressed as % with respect to the negative control (CTRL).

### 5.4. Mortality Assay

The mortality of nauplii stage I and adults after exposure to PLTX (0.1–10.0 nM) was expressed as the percentage of dead organisms with respect to the total number of the animals in each sample. The mortality of nauplii stage I was evaluated on 5 nauplii/well suspended in 200 µL of seawater, using 96-well plates, while that of adults was evaluated using 24-well plates with 10 adults/well in 1.5 mL of seawater. Adults were fed with solid food 24 h before the assay and, in the case of time-course exposure up to 72 h, also 24 h after it began. On the contrary, nauplii did not receive feed. According to Zulkifli et al. [61], animals were considered dead if they did not show any movement for 10 s under the stereomicroscope observation (Kyowa; Tokyo, Japan) at a 3× magnification (nauplii) or 1× magnification (adults).

### 5.5. Reactive Oxygen Species Quantitation

To assess oxidative stress in *A. franciscana* adults after exposure to PLTX for 12 h, reactive oxygen species (ROS) were measured as previously reported [41,62]. The samples were prepared by collecting five organisms for each group of treatment, which were washed three times with 100 mM Tris-HCl, pH 7.5, and homogenized for 15 s in 1 mL of the same buffer, using an immersion sonicator (Ultrasonic processor UP50H; Hielscher; Teltow, Germany). After centrifugation at 12,000× *g* for 15 min at 4 °C, the supernatant was collected and preserved at −80 °C. ROS production was evaluated using the 2′,7′-dichlorofluorescin diacetate (DCFDA) assay as previously reported [41,62]. Briefly, using 96-well plates, 160 µL of phosphate buffered saline (PBS), 20 µL of 400 µM DCFDA (Sigma-Aldrich; Milan, Italy), and 20 µL of supernatant were incubated at 37 °C for 30 min in the dark. The fluorescence was read at 485 nm excitation and 520 nm emission, using a microplate reader (Fluorocount; Packard; Germany), and the results were expressed as relative fluorescence units (RFU) normalized on milligrams of proteins for each sample, measured using a NanoDrop 2000 instrument (Thermo Scientific; Milan, Italy).

### 5.6. Preparation of A. franciscana Samples for the Quantitation of the Enzymes’ Activity

In a 96-well plate, 50 *Artemia* adults were exposed to PLTX (0.1–10 nM) for 12 h and subsequently processed as previously described [41,63]: the organisms were washed three times with phosphate buffer (composed of 50 mM Na_2_HPO_4_-7H_2_O and NaH_2_PO_4_H_2_O, pH 7.0) containing EDTA (5 mM), sonicated in 240 µL of phosphate buffer (50 mM, pH 7.0) for 15 s, and centrifuged at 15,000× *g* for 25 min at 4 °C. After protein quantitation using the NanoDrop 2000 instrument (Thermo Scientific; Milan, Italy), the supernatants were collected and preserved at −80 °C, until the measurement of the enzymes’ activity.

### 5.7. Glutathione S-Transferase Activity

Glutathione S-transferase (GST) activity in *A. franciscana* adults exposed to PLTX for 12 h was quantified using a reaction mixture of 1.3 mM reduced L-glutathione (Sigma-Aldrich; Milan, Italy) and 1.3 mM 1-chloro-2,4-dinitrobenzene (CDNB; Sigma-Aldrich; Milan, Italy) in a potassium phosphate buffer (composed of 100 mM K_2_HPO_4_ and KH_2_PO_4_, pH 6.5) [41,63]. In each well of a 96-well plate, 75 µL of the mixture was added to 25 µL of each sample and the absorbance was read immediately at 304 nm, and subsequently every 30 sec for 3 min, using a microplate reader (FLUOstar Omega, BMG Labtech; Ortenberg, Germany). The blank was prepared in the same way, replacing the probe (CDNB) with the buffer.

### 5.8. Catalase Activity

Catalase (CAT) activity in *A. franciscana* adults exposed to PLTX for 12 h was quantified as previously described by Zhao et al. [64] on earthworms, adapting the assay for *Artemia*. Briefly, 20 µL of each sample was added to 980 µL of 10 mM H_2_O_2_ (Sigma-Aldrich; Milan, Italy) in 50 mM phosphate buffer, pH 7.0 (composed of Na_2_HPO_4_ and NaH_2_PO-4H_2_O), in a quartz cuvette. The absorbance was read immediately at 240 nm, and every 30 s for 3 min, using a UV-Vis spectrophotometer (Thermo Spectronic Helios Y; Thermo Scientific; Milan, Italy).

### 5.9. Peroxidase Activity

Peroxidase (POD) activity in *A. franciscana* adults exposed to PLTX for 12 h was quantified according to the protocol reported by Zhao et al. [64]. In each well of a 96-well plate, 50 µL of *A. franciscana* sample was added to 100 µL of a reaction mixture containing phosphate buffer (100 mM pH 6.0, composed of Na_2_HPO_4_ and NaH_2_PO-4H_2_O), 3% H_2_O_2_ (Sigma-Aldrich; Milan, Italy), and 100 mM guaiacol (Sigma-Aldrich; Milan, Italy). The absorbance was read immediately at 470 nm, and subsequently every 30 s for 3 min, using a microplate reader (FLUOstar Omega, BMG Labtech; Ortenberg, Germany).

### 5.10. Superoxide Dismutase Activity

According to Zhao et al. [64], the activity of superoxide dismutase (SOD) was quantified by measuring the enzyme ability to inhibit the photochemical reduction of nitro blue tetrazolium (NBT). The assay was performed using 96-well plates, adding to each well 25 µL of sample and 75 µL of a reaction mixture. The reaction mixture was composed of 50 mM phosphate buffer, pH 7.8 (composed of Na_2_HPO_4_ and NaH_2_PO-4H_2_O), containing 140 mM L-methionine (Sigma-Aldrich; Milan, Italy), 750 µM NBT (Sigma-Aldrich; Milan, Italy), 20 µM Riboflavin (Sigma-Aldrich; Milan, Italy), and 100 µM Na_2_EDTA (Sigma-Aldrich; Milan, Italy). Blank was created by replacing 25 µL of each sample with the same volume of phosphate buffer. The 96-well plate was incubated at room temperature for 30 min under a 900-lumen lamp to allow the photochemical reaction, then the absorbance was read at 560 nm using a microplate reader (FLUOstar Omega; BMG Labtech; Ortenberg, Germany).

### 5.11. Cholinesterase Activity

Cholinesterase (ChE) activity was measured following the procedure reported by Jemec et al. [63]. In each well of a 96-well plate, 50 µL of *A. franciscana* sample was diluted with 50 µL of a reaction mixture consisting of 100 mM potassium phosphate buffer, pH 7.4, containing 2 mM acetylthiocholine iodide (Sigma-Aldrich; Milan, Italy) and 1 mM 2,2-dinitro-5,5-dithiobenzoic acid (DTNB; Sigma-Aldrich; Milan, Italy). The reaction mixture of blank was prepared replacing DTNB with the buffer. The absorbance of the sample was measured at 405 nm, every minute for 3 min, with a microplate reader (FLUOstar Omega; BMG Labtech; Ortenberg, Germany).

### 5.12. Enzymes’ Activity Data Analysis

The enzymes’ (GST, CAT, POD, and ChE) activities in *Artemia* samples were expressed as enzyme units (*EU*), calculated by the following formula:

$$EU=\frac{\frac{\Delta Absorbance\;}{min}}{\varepsilon *l}*\frac{V\;total}{V\;supernatant}$$

where *ε* values were 9600 M^−1^ *cm^−1^ for GST, 43.6 M^−1^ *cm^−1^ for CAT, 0.0266 M^−1^ *cm^−1^ for POD, and 13,600 M^−1^ *cm^−1^ for ChE activity assays. The enzymes’ activity was normalized on the mg of proteins (*EU*/mg proteins) of each sample. The relevant measure units were nmol/min for GST and ChE, µmol/min for CAT, and mmol/min for POD.

One *EU* of SOD was defined as the amount of enzyme needed to inhibit the photochemical reduction of NBT by 50%, and SOD activity was reported as *EU*/mg of proteins.

### 5.13. Protein Content

The proteins in the supernatant of each *A. franciscana* sample prepared to determine the enzymes’ activity and ROS production were quantified using a NanoDrop 2000 (Thermo Scientific; Milan, Italy) at 280 nm.

### 5.14. Statistical Analysis

All the results are expressed as mean ± standard errors of the mean (SE) of at least three independent experiments. LC_50_ values were calculated by variable slope (four-parameter) non-linear regression at a statistical confidence interval of 95%, using the GraphPad Prism version 6.

Depending on the biological assays, data were analysed by a one or two-way analysis of variance (ANOVA) and Bonferroni post-test, or by the Student’s *t*-test, using the GraphPad Prism software version 6. Significant differences were considered as *p* < 0.05.

## Supplementary Materials

The following supporting information can be downloaded at: https://www.mdpi.com/article/10.3390/md20020081/s1, Video S1: video showing *Artemia franciscana* adults’ movement after 12 h exposure to PLTX (0.1–10.0 nM) in comparison to the control group not exposed to the toxin. The video was acquired on an inverted optical microscope at 4× magnification.
